# Supplementary material for: Single amino acid change alters specificity of the multi-allelic wheat stem rust resistance locus SR9
Source: Nat Commun. 2023 Nov 14;14:7354. doi: 10.1038/s41467-023-42747-9 (PMC10645757; doi:10.1038/s41467-023-42747-9)
Supplement: Supplementary file 1 — Supplementary Information [file 41467_2023_42747_MOESM1_ESM.pdf]

Single amino acid change alters specificity of the multi-allelic wheat stem rust resistance locus *SR9*

Zhang *et al.*

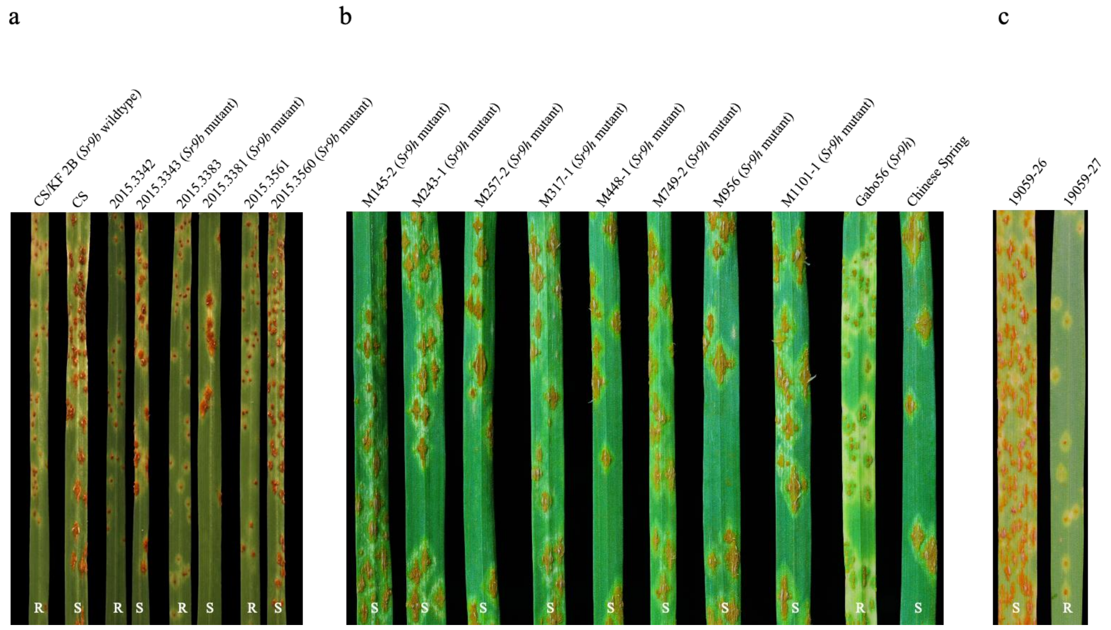

Supplementary Figure 1. EMS mutants used for isolating or validating *Sr9b*, *Sr9h*, *Sr9e_h2* gene candidates.

a. Seedling infection types of *Sr9b* wildtype CS/KF 2B, CS, three mutants and their resistant sibs in response to *Pgt* race 126-5,6,7,11 (culture no. 217). b. Seedling infection types of *Sr9h* wildtype Gabo 56, CS and eight susceptible mutants derived from Gabo 56 in response to *Pgt* race TTKSK (isolate 04KEN156/04). c. Seedling infection types of lines 19059-26 and 19059-27 from the cross between two Kronos mutant lines T4-3163 (carrying non-functional *Sr9e_h2*) and T4-3102 (carrying non-functional *Sr13*) in response to *Pgt* race TRTTF (06YEM34-1). “R” for resistance and “S” for susceptible.

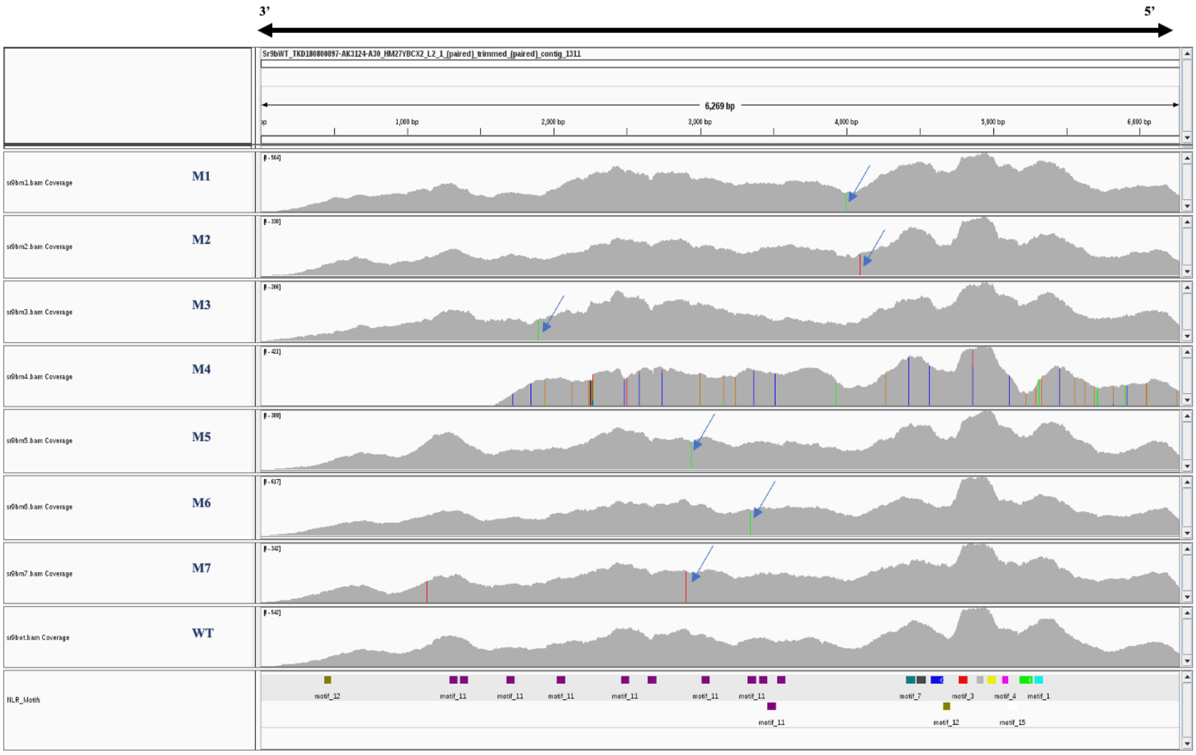

Supplementary Figure 2. IGV snapshot for *Sr9b* gene candidate captured from MutRenSeq pipeline.
Six of the seven mutants of *Sr9b* carried SNP changes (indicated by blue arrows) in comparison with the wildtype. The image illustrates sequence read numbers (Y-axis, read number range shown in the top left corner) and position in the candidate gene contigs (X-axis). The contig orientation is shown on top of each figure. Coloured rectangles represent conserved NLR motifs identified by NLR-Parser.

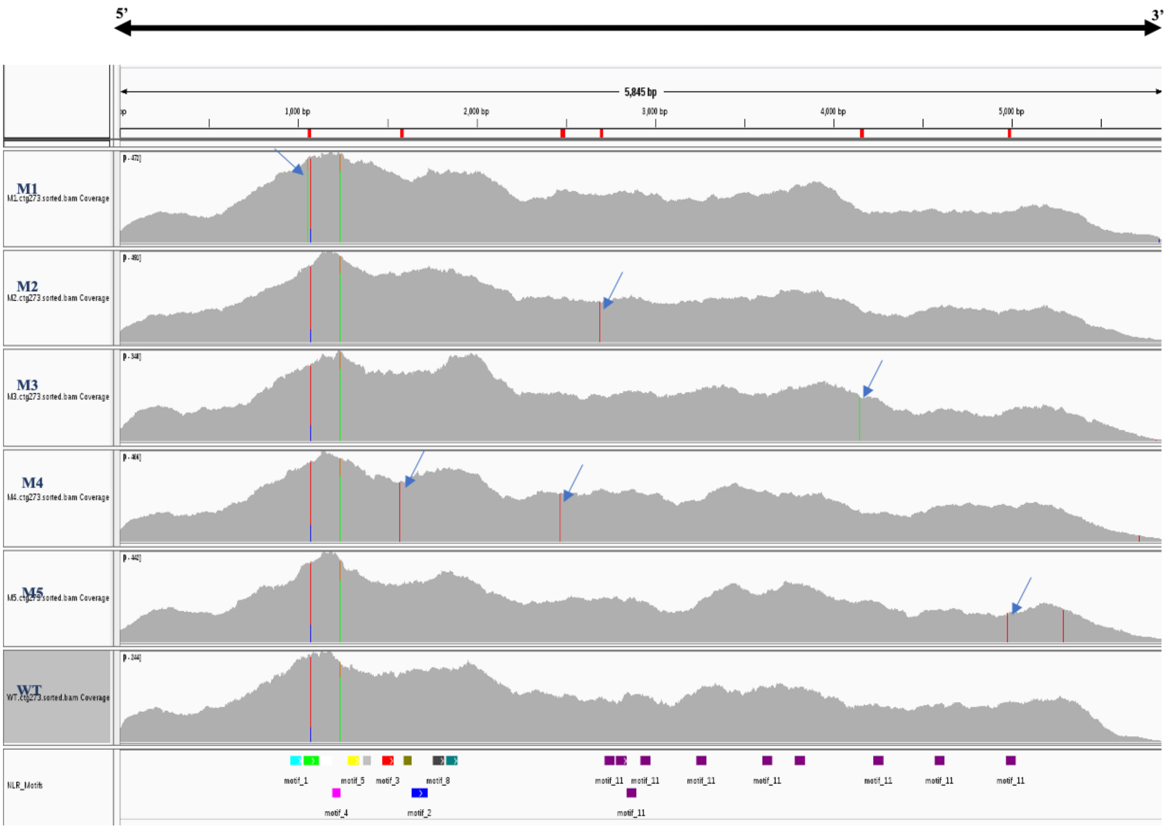

Supplementary Figure 3. IGV snapshot for *Sr9e_h1* gene candidate captured from MutRenSeq pipeline.

All five mutants of *Sr9e_h1* carried SNP changes (indicated by blue arrows) in comparison with the wildtype cv. Vernstein. The image illustrates sequence read numbers (Y-axis, read number range shown in the top left corner) and position in the candidate gene contigs (X-axis). The contig orientation is shown on top of each figure. Coloured rectangles represent conserved NLR motifs identified by NLR-Parser.

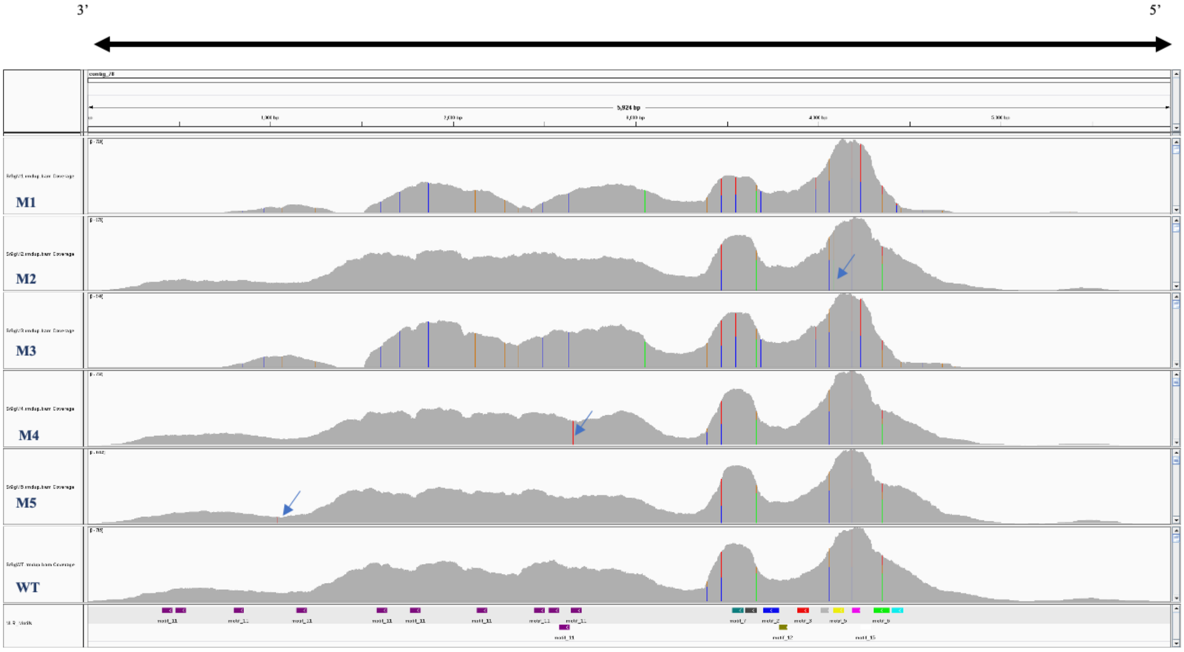

Supplementary Figure 4. IGV snapshot for *Sr9g* gene candidate captured from MutRenSeq pipeline.

Three of the five *Sr9g* mutants showed SNP polymorphisms (indicated by blue arrows) compared to wildtype CS/Marquis 2B. The image illustrates sequence read numbers (Y-axis, read number range shown in the top left corner) and position in the candidate gene contigs (X-axis). The contig orientation is shown on top of each figure. Coloured rectangles represent conserved NLR motifs identified by NLR-Parser.

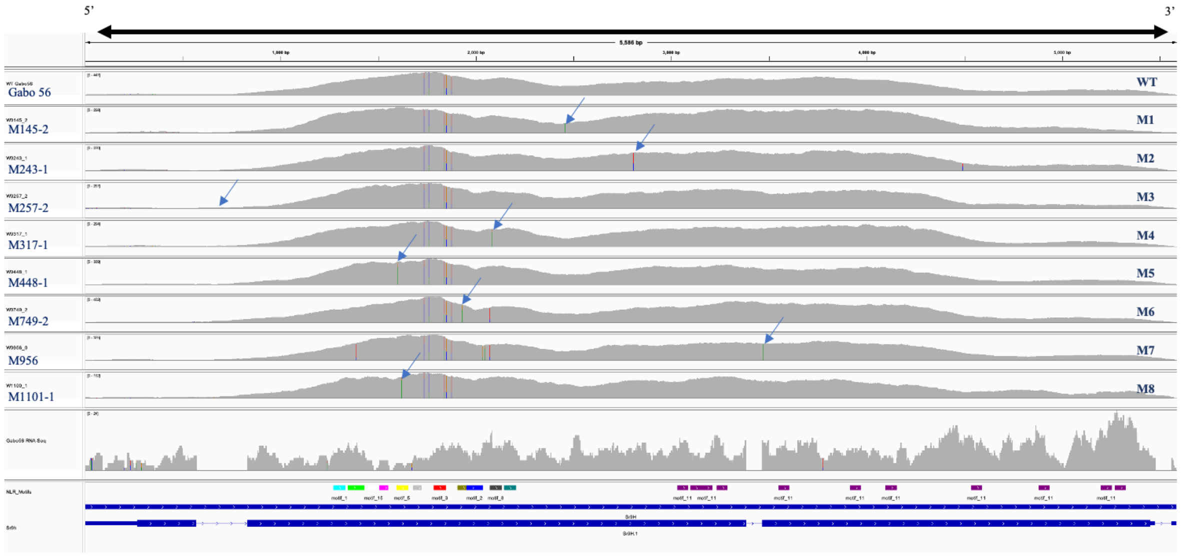

Supplementary Figure 5. IGV snapshot for *Sr9h* gene candidate captured from MutRenSeq pipeline.

Assembly of MutRenSeq contig_4073_1 showing the SNPs (blue arrows) in seven of the eight mutants within the coding region of the candidate *Sr9h* gene. The image illustrates sequence read numbers (Y-axis, read number range shown in the top left corner) and position in the candidate gene contigs (X-axis). The contig orientation is shown on top of each figure. Coloured rectangles represent conserved NLR motifs identified by NLR-Parser.

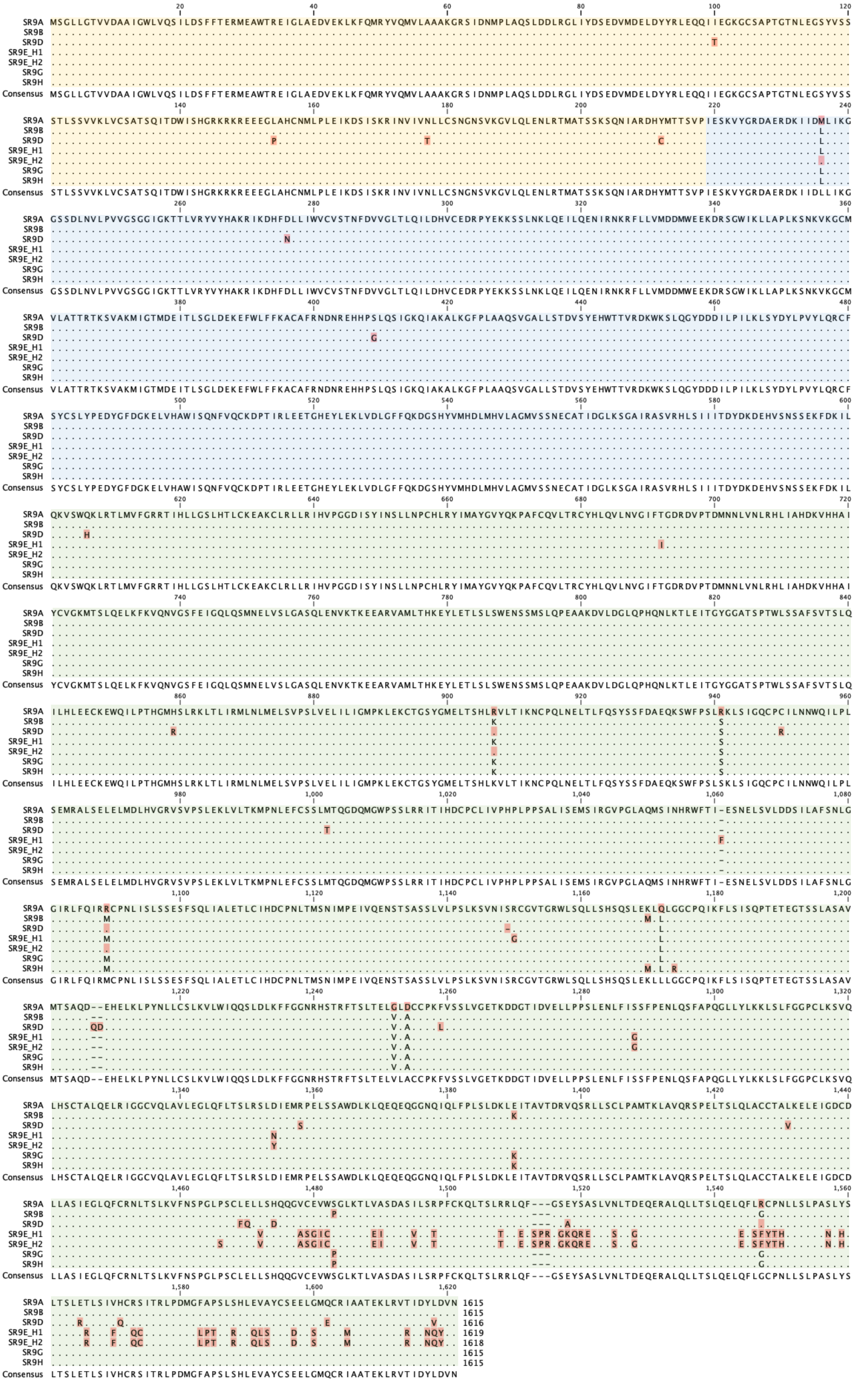

Supplementary Figure 6. Protein sequence alignment of the seven SR9 proteins. All polymorphic residues are shaded with red. Different domains are shaded with light yellow (CC domain), light blue (NB-ARC domain), and light green (LRR domain).

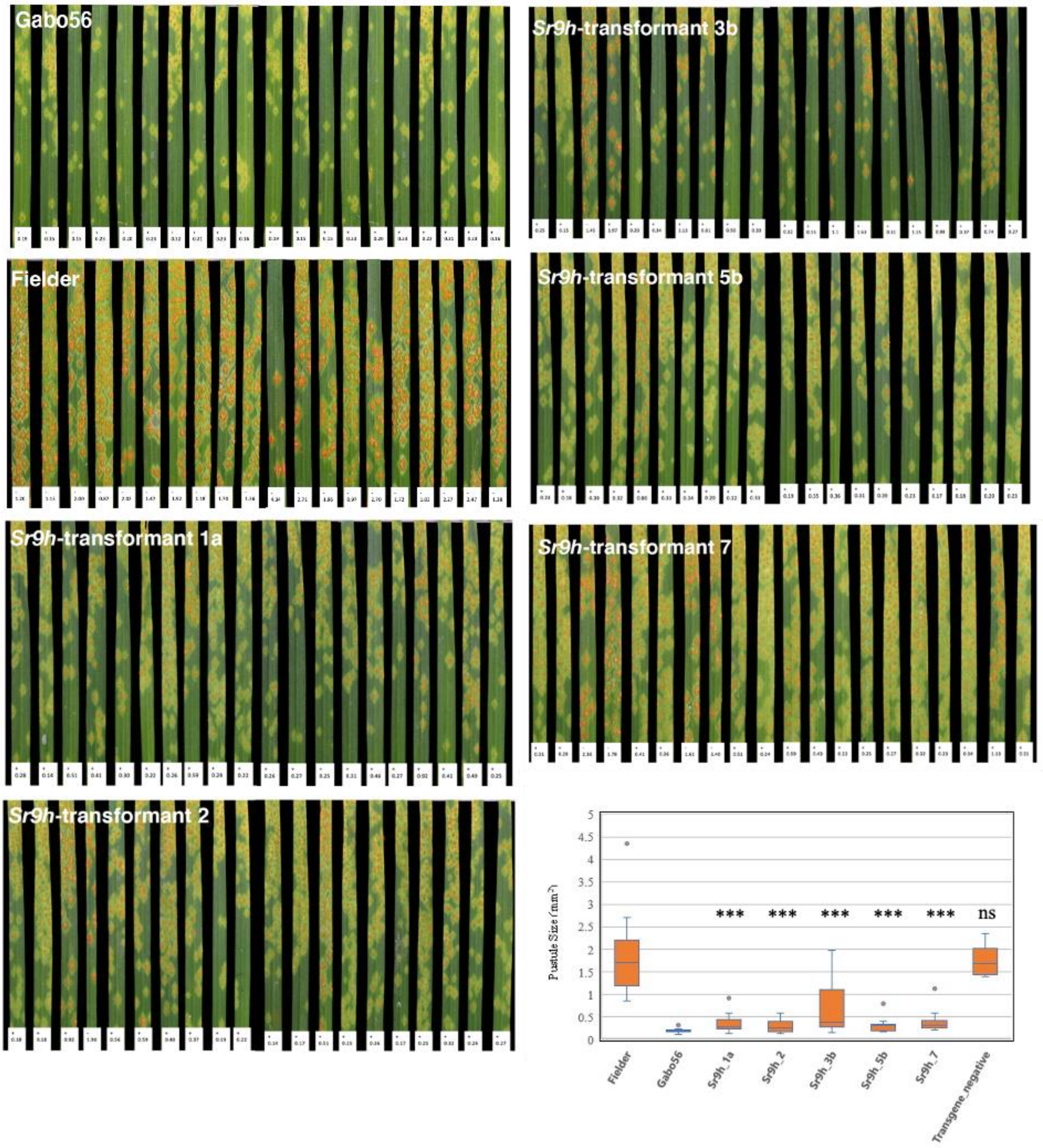

Supplementary Figure 7. Seedling infection types of Gabo 56 (*Sr9h*), Fielder and five T_1 families of *Sr9h* transformants in a Fielder background in response to *Pgt* race TTKSK. Sr9h-1a, Sr9h-2, Sr9h-3b, Sr9h-5b, and Sr9h-7 are transgenic families with the *Sr9h* gene in Fielder background. The 'Transgene negative' line included a total of six progeny from families Sr9h-2, Sr9h-3b, and Sr9h-7 that did not possess the *Sr9h* transgene based on PCR ("+" indicates presence and "-" indicates absence). Average pustule size for response to *Sr9h*-avirulent *Pgt* race TTKSK (Ug99) across eight wheat lines. Significant differences using *t* tests relative to response on 'Fielder' wheat were indicated by '***' ($p<0.001$) or 'ns' (not significant, $p>0.05$). Wheat line 'Gabo 56' possesses *Sr9h*, but is not the same genetic background as the other lines ('Fielder' background), therefore was not tested. Source data are provided as a Source Data file.

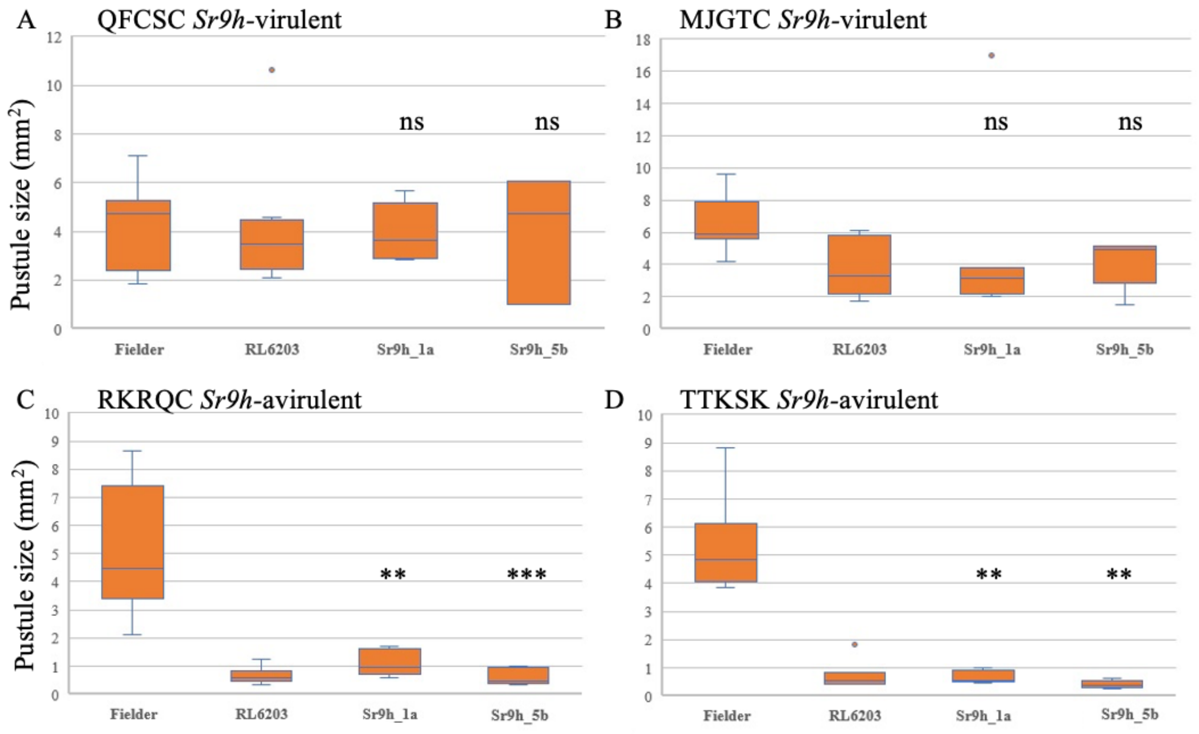

Supplementary Figure 8. Average pustule size for response to *Pgt* races QFCSC, MJGTC, RKRQC and TTKSK across Fielder, RL6203 and two Fielder transgenic lines with *Sr9h*. Average pustule size for response to *P. graminis* f. sp. *trici* races QFCSC (A), MJGTC (B), RKRQC (C) and TTKSK (D) across four wheat lines: Fielder, RL6203 (*Sr9h* monogenic line), and two Fielder transgenic lines with *Sr9h* (Sr9h-1a and Sr9h-5b). Races QFCSC and MJGTC are virulent to *Sr9h* whereas RKRQC and TTKSK are virulent. Significant differences using *t* test relative to response on 'Fielder' wheat were indicated by '**' (p<0.01), '***' (p<0.001), or 'ns' (not significant, p>0.05). Wheat line 'RL6203' possesses *Sr9h*, but is not the same genetic background as the other lines ('Fielder' background), therefore was not tested. Source data are provided as a Source Data file.

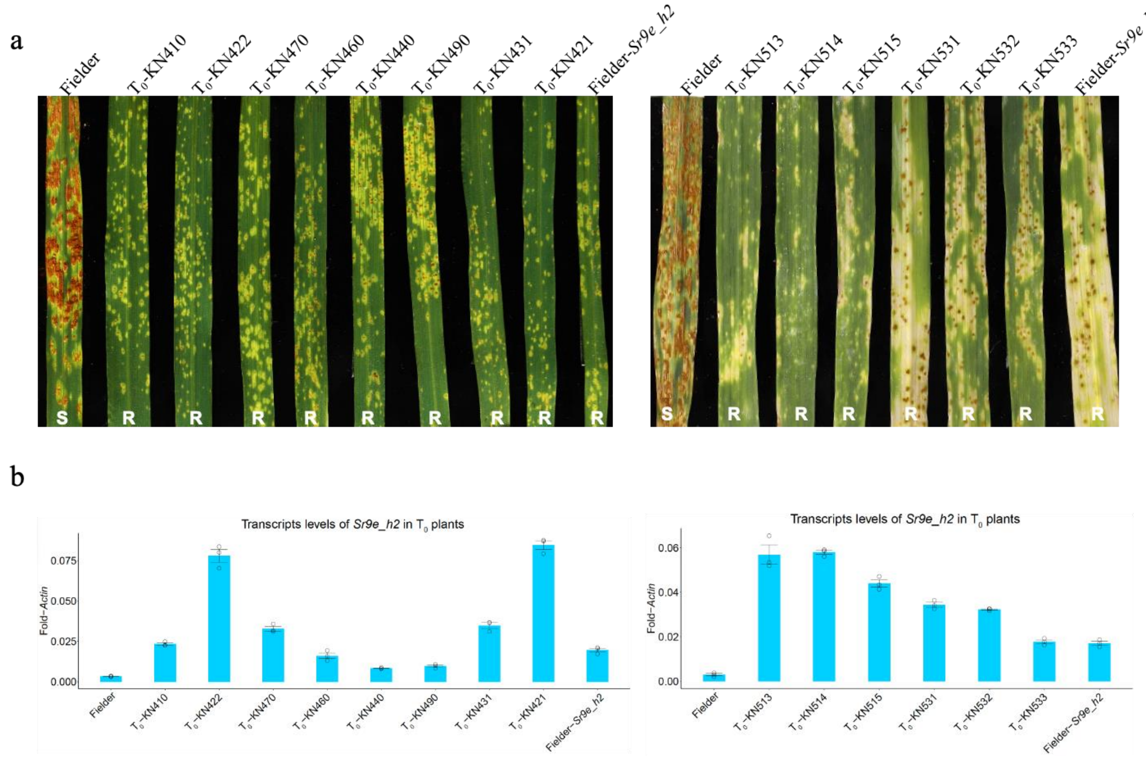

Supplementary Figure 9. Gene *Sr9e_h2* confers resistance when transferred into the susceptible Fielder.

a. Reactions to *Pgt* race 34MKGQM (isolate 20IAL06) in Fielder control, 14 independent transgenic T_0 plants, and introgression line Fielder-*Sr9e_h2* (PI 700734) (BC2F4, positive control). S, susceptible; R, resistant. 1, Fielder control; 2-15, transgenic plants T_0-KN410, T_0-KN422, T_0-KN470, T_0-KN460, T_0-KN440, T_0-KN490, T_0-KN431, T_0-KN421, T_0-KN513, T_0-KN514, T_0-KN515, T_0-KN531, T_0-KN532, and T_0-KN533; 16, introgression line Fielder-*Sr9e_h2* (PI 700734, Fielder-*Sr9e_SrKN*). b. Transcript levels of *Sr9e_hb* based on three technical replicates from a single plant (as in a). Transcript levels are expressed as fold-*ACTIN* using the $2^{-\Delta CT}$ method. Error bars are standard errors of the mean. Source data are provided as a Source Data file.

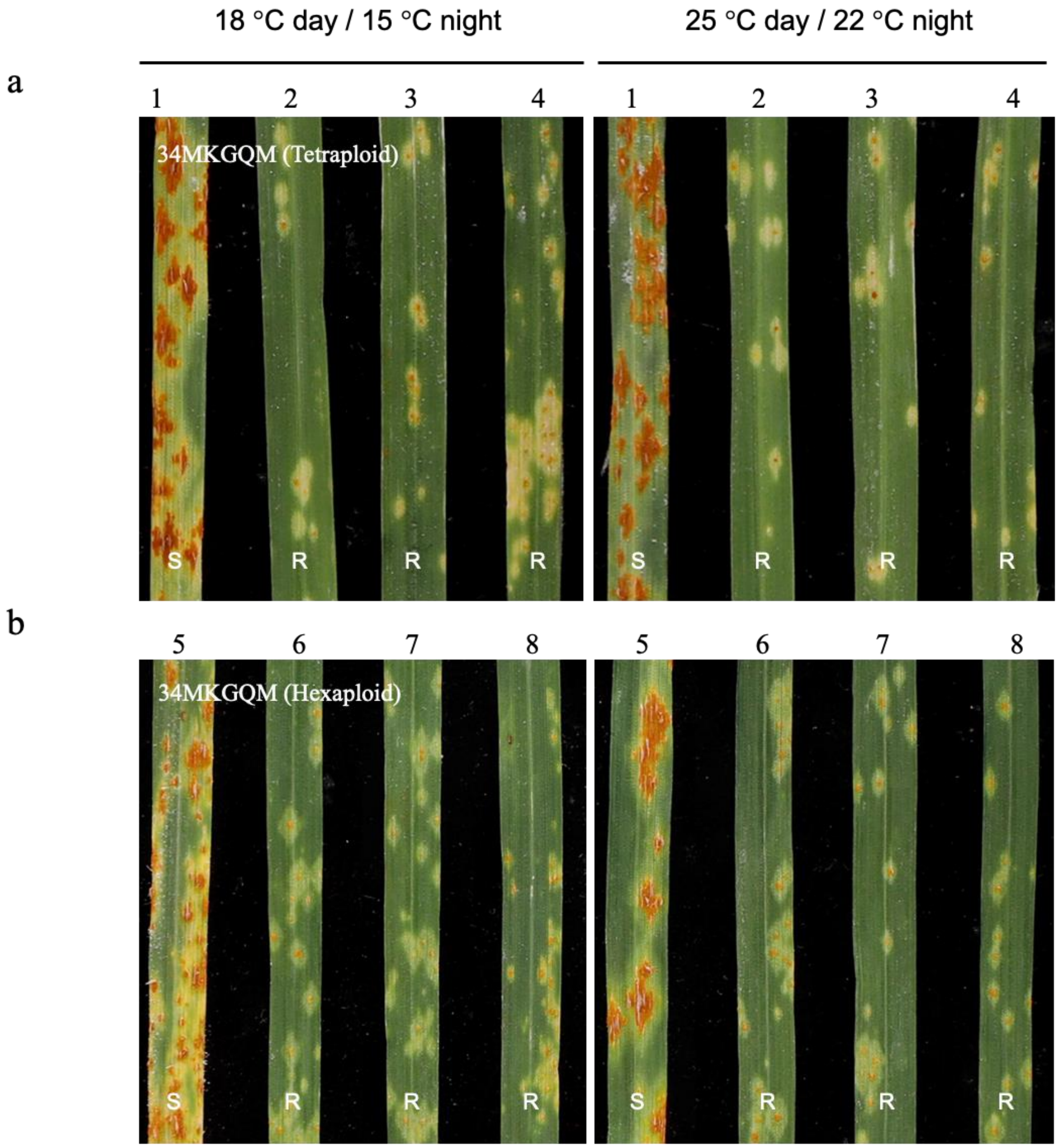

Supplementary Figure 10. Reactions to *Pgt* race 34MKGQM (isolate 20IAL06) at two temperature regimes (18 °C day / 15 °C night and 25 °C day / 22 °C night).
a. Infection types on Rusty (1) and *Sr9e_h2* monogenic line Td31-5R (2-4) inoculated with race 34MKGQM. b. Infection types on Fielder (5) and *Sr9e_h2* introgression line Fielder-*Sr9e_h2* (PI 700734) (6-8) inoculated with race 34MKGQM. Both experiments were performed under long days (16 h light / 8 h dark). Leaves were photographed when the control lines (Rusty and Fielder) were fully susceptible. R, resistant; S, susceptible.

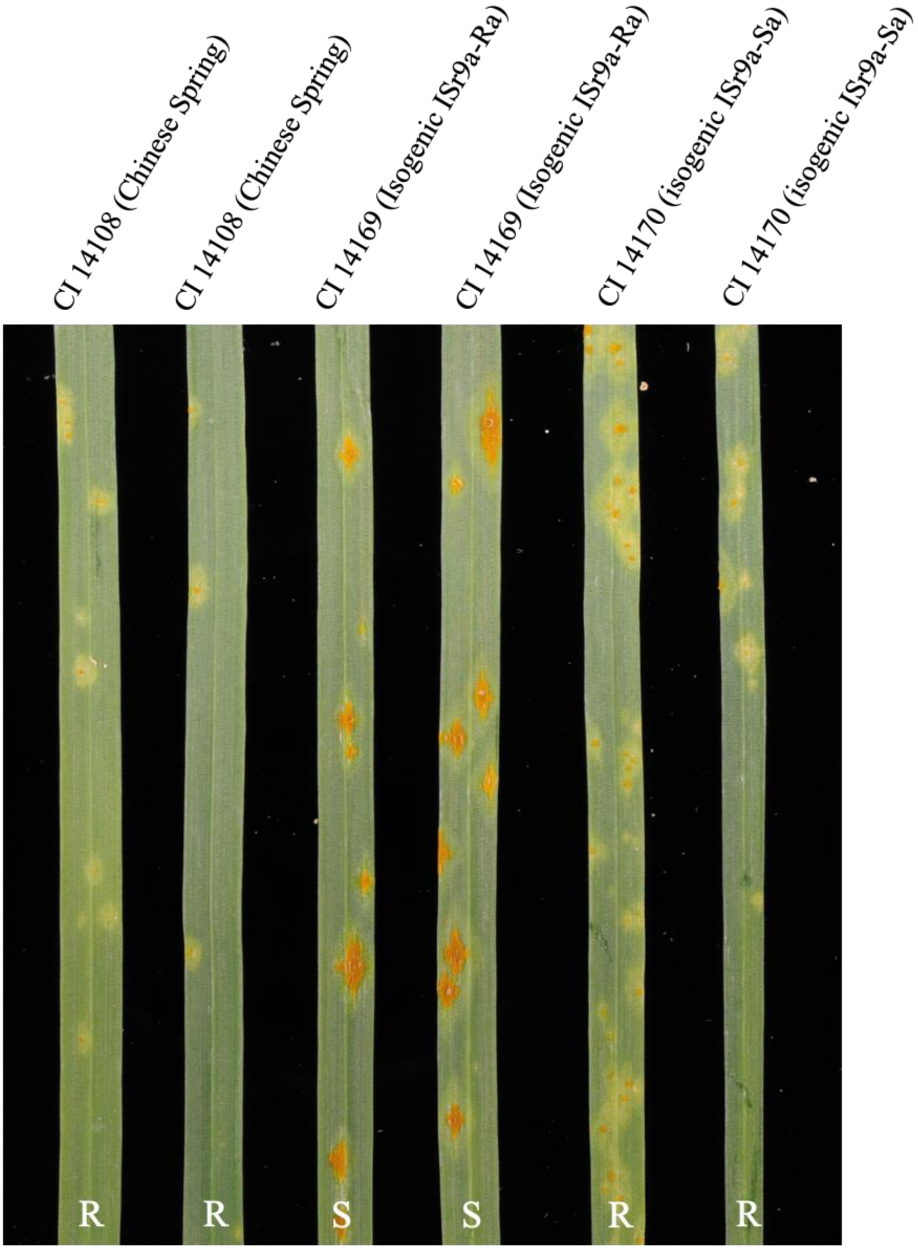

Supplementary Figure 11. Stem rust responses of Chinese Spring (CI 14108) and near-isogenic lines (NILs), ISR9a-Ra and ISR9a-Sa, infected by *Pgt* culture '111X36'. "R" stands for resistance and "S" for susceptible.

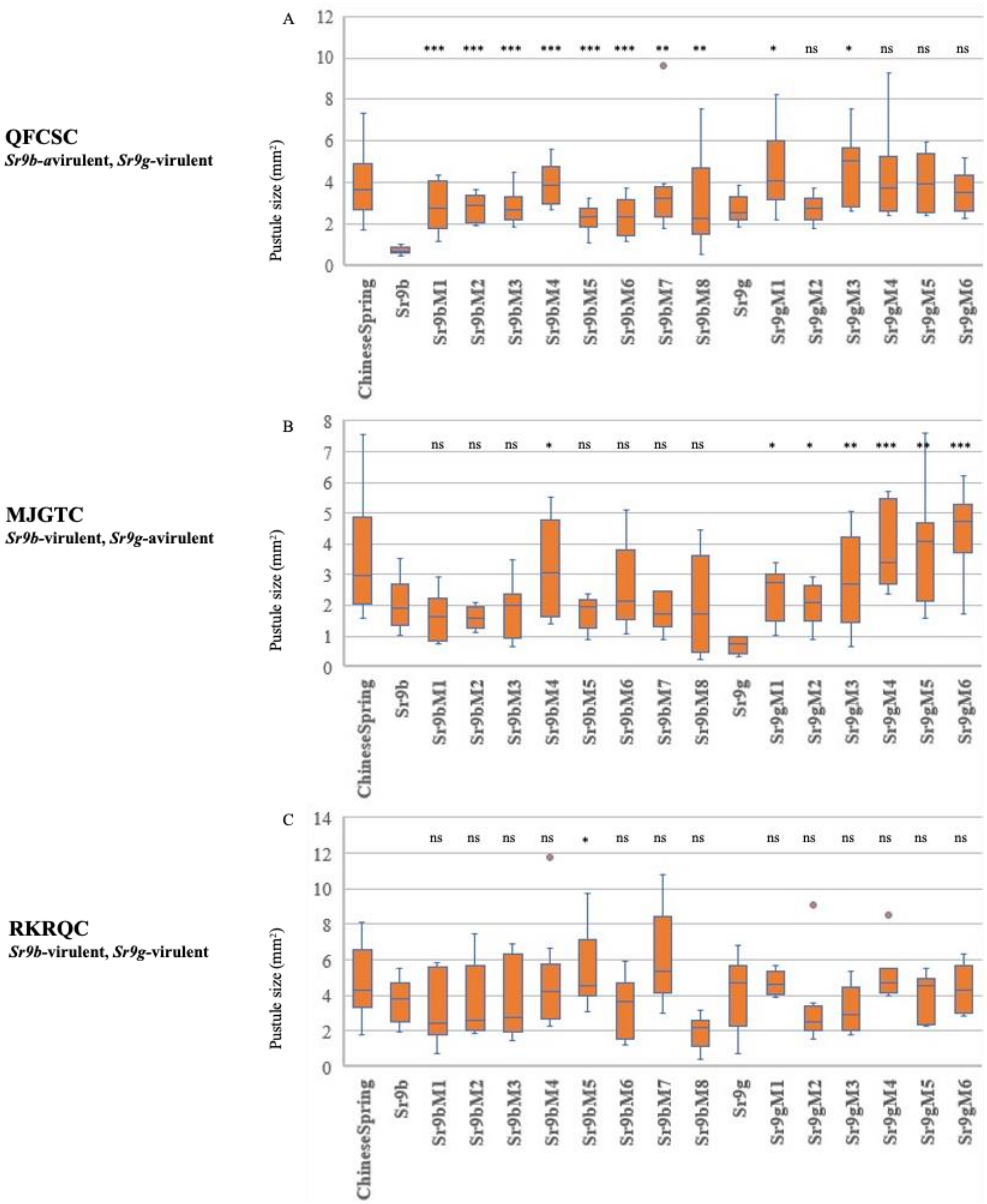

Supplementary Figure 12. Average pustule size for response to *Pgt* races QFCSC, MJGTC, and RKRQC across 17 wheat lines including Chinese Spring, CS/KF 2B (*Sr9b*), CS/Mq 2B (*Sr9g*), and mutant lines derived from both CS/KF 2B and CS/Mq 2B.

Race QFCSC (A) is *Sr9b*-avirulent and *Sr9g*-virulent. Race MJGTC (B) is *Sr9b*-virulent and *Sr9g*-avirulent. Race RKRQC (C) is virulent to both *Sr9b* and *Sr9g*. The *Sr9b* mutant lines were tested for significant differences compared to CS/KF 2B (*Sr9b*) whereas the *Sr9g* mutant lines were tested for significant diffrences compared to CS/Mq 2B (*Sr9g*). Significant differences using *t* tests were indicated by '*' (p<0.05), '**' (p<0.01), '***' (p<0.001), or 'ns' (not significant, p>0.05). Source data are provided as a Source Data file.

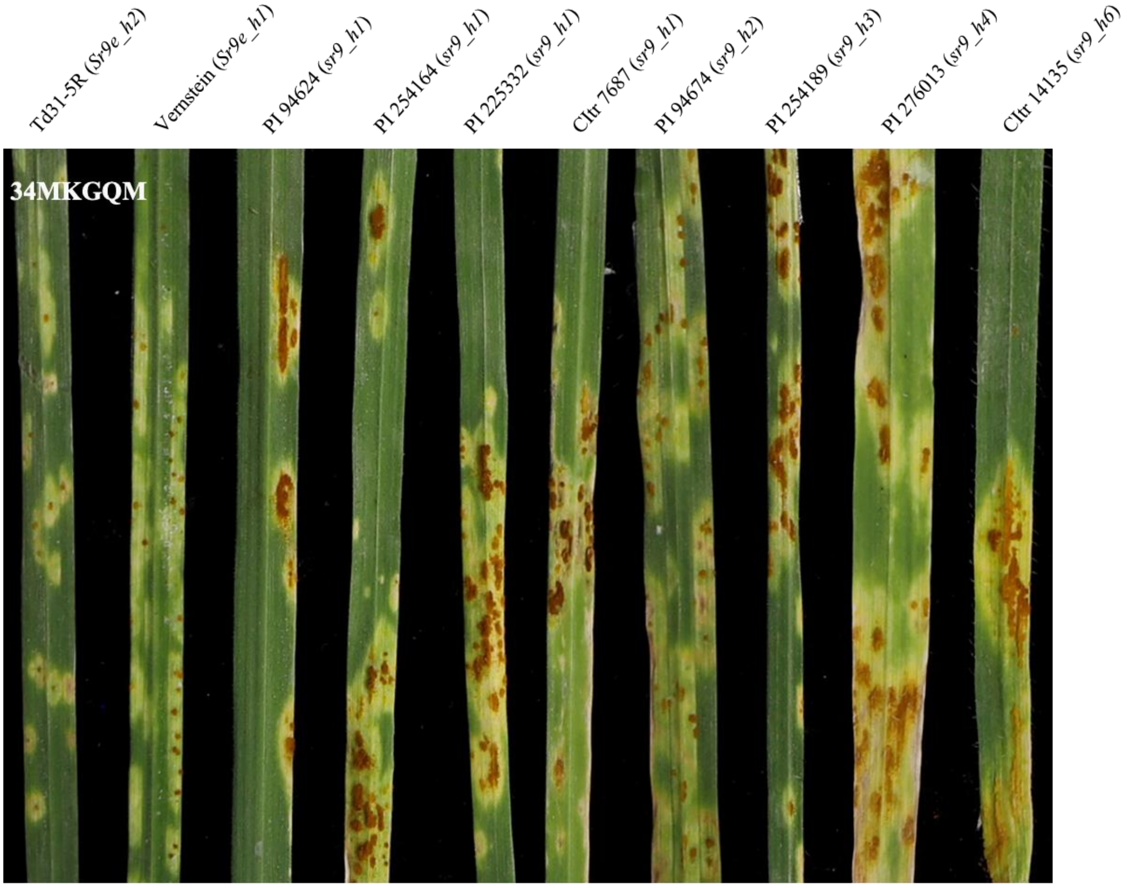

Supplementary Figure 13. Reactions to *Pgt* race 34MKGQM in lines carrying haplotypes *Sr9e_h2*, *Sr9e_h1*, *sr9_h1*, *sr9_h2*, *sr9_h3*, *sr9_h4*, and *sr9_h6* at 22 °C with a 16 h light photoperiod.

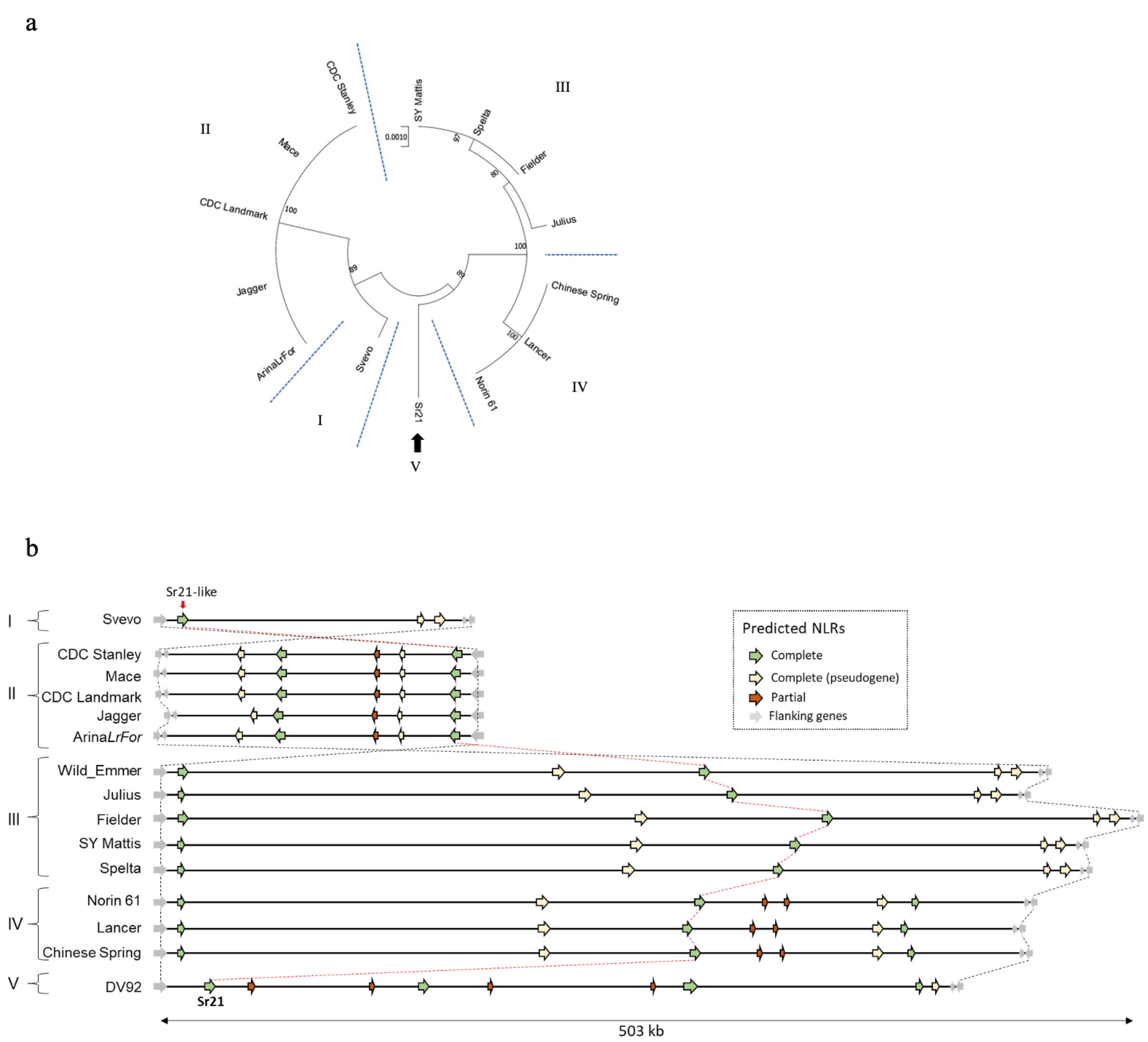

Supplementary Figure 14. NLR haplotype analysis of the wheat pangenome at the corresponding SR21 locus on chromosome 2A.

a. Phylogenetic grouping using the genomic sequence of *Sr21* derived from *T. monococcum* (vertical black arrow) and its closest homologs in sequenced *T. aestivum* and *T. turgidum* (Svevo) wheat genomes. b. Schematic of predicted NLRs in different *T. aestivum* and *T. turgidum* (Svevo and wild emmer) lines at the *Sr21* homologous region as well as the sequenced BAC contig of the cloned *Sr21* donor line DV92. NLRs are represented by coloured arrows according to NLR Annotator results as complete NLRs (green), complete pseudogenes (yellow) or partial NLRs (red). Some structural variations were observed with the closest Sr21 homolog represented by either the first or third NLR of the cluster (red connecting line). Haplotype II has an inversion of the whole genomic region but is overall conserved relative to the other genomes. The numbering I to V highlights phylogenetic subgroups (a) with conserved NLR clustering (b).

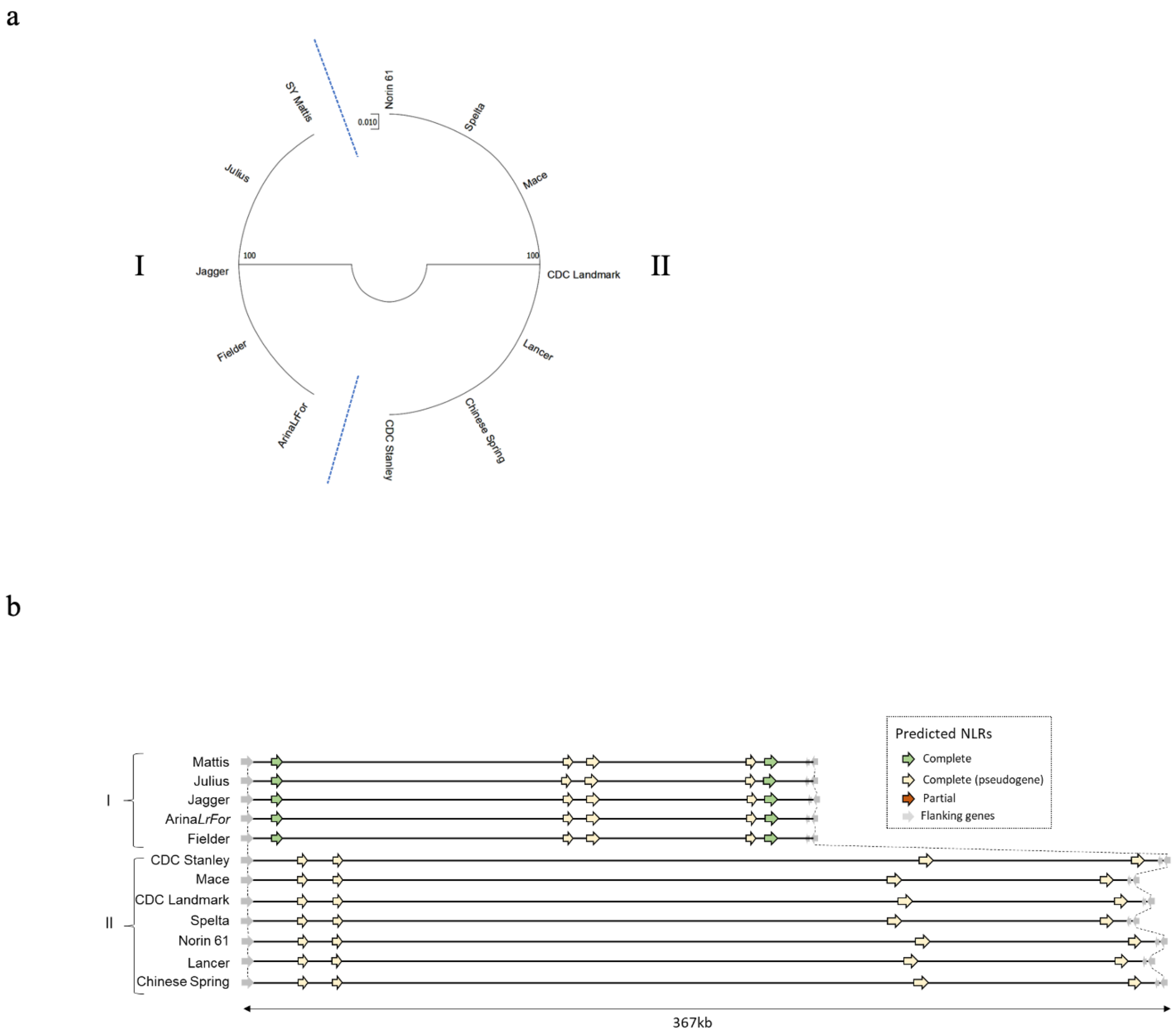

Supplementary Figure 15. NLR haplotype analysis of the wheat pangenome at the SR9 homologous region on chromosome 2D.

a. A phylogenetic grouping using genomic sequences of *SR9* closest homologs in sequenced wheat genomes. b. Schematic of predicted NLRs in wheat lines at the SR9 homologous region. NLRs are represented by coloured arrows according to NLR Annotator output as complete NLRs (green), complete pseudogenes (yellow), or partial NLRs (red). The numbering I to II highlights phylogenetic sub-groups (a) having a conserved NLR clustering (b).

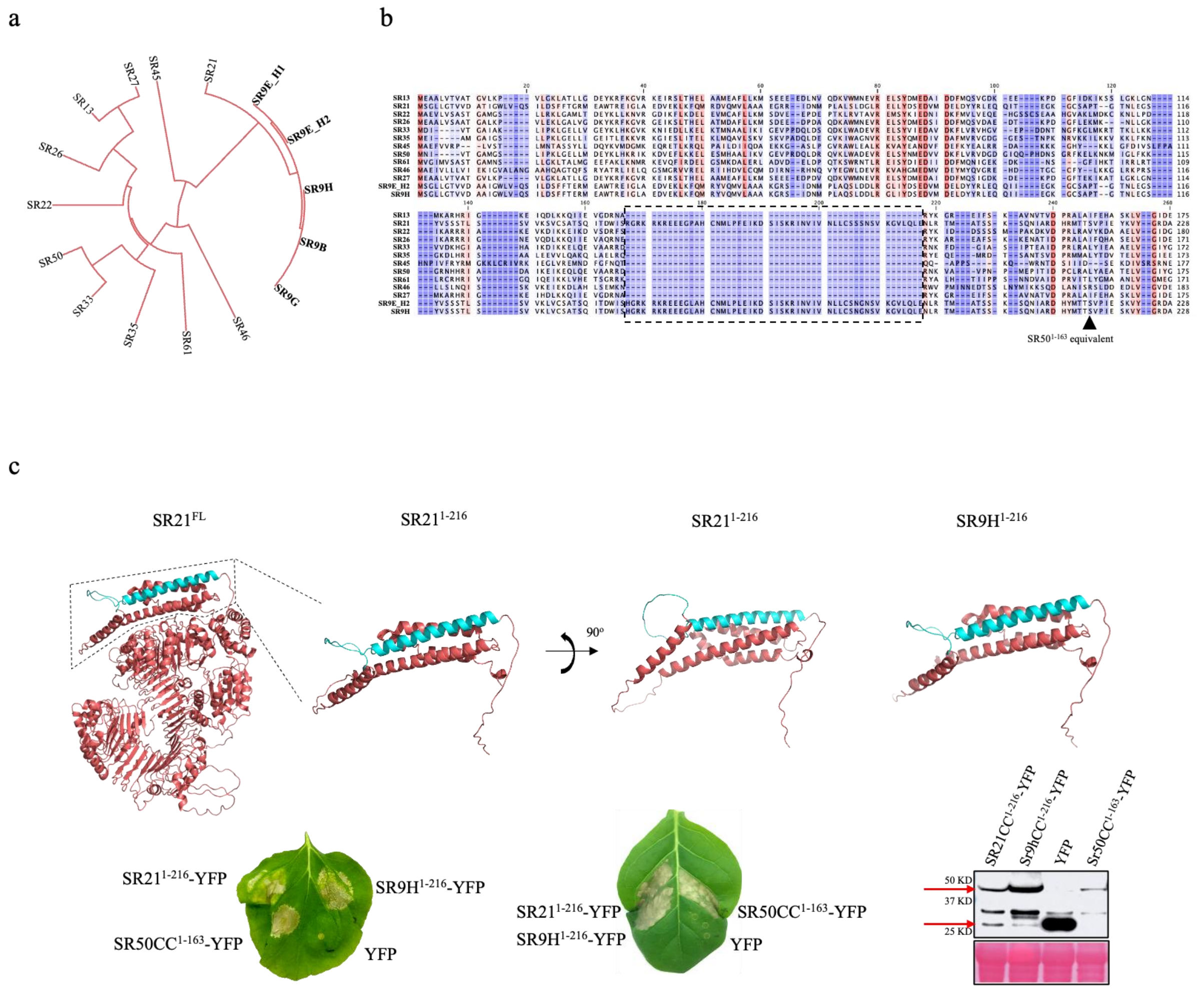

Supplementary Figure 16. Protein sequence alignment and functional analysis of the SR9H and SR21 N terminal domains.

a. Stem rust resistance protein phylogenetic tree constructed using Expresso; b. Alignment of the N-terminal coiled-coil domain regions of stem rust resistance proteins. A 51 aa sequence unique to SR9 and SR21 is indicated within the black dotted lined frame. A solid black arrow indicates the position 163 in SR50 that defines an autoactive fragment of this protein and corresponds to amino acid 216 in SR9 and SR21; **c**. Predicted protein structural model of SR21 by DeepMind AlphaFold tool v2.0. The 51 aa inserted sequences are coloured in cyan. Both SR21$^{1-216}$ and SR9H$^{1-216}$ are able to trigger cell death in *N. benthamiana* and *N. tabacum.* SR50CC$^{1-163}$ and YFP are used as positive and negative control, respectively. Immunoblot of SR21$^{1-216}$ and SR9H$^{1-216}$ YFP-tagged proteins detected with anti-GFP. Similar results were obtained in three independent experiments. Source data are provided as a Source Data file.

a

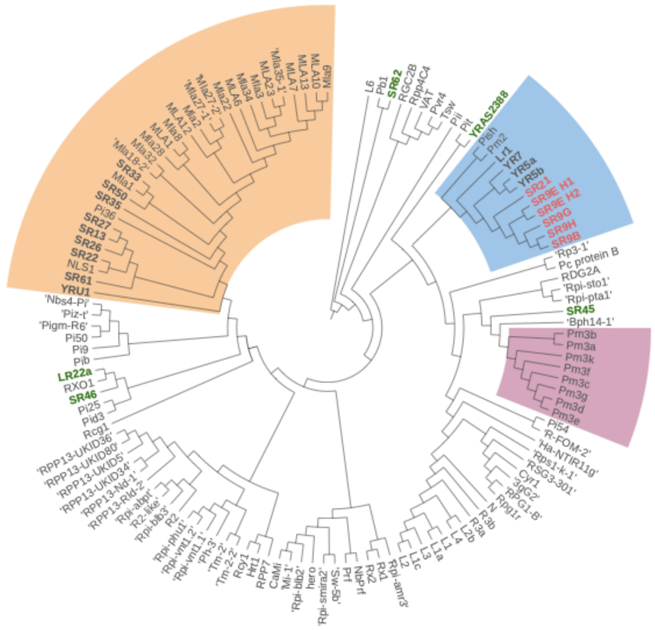

b

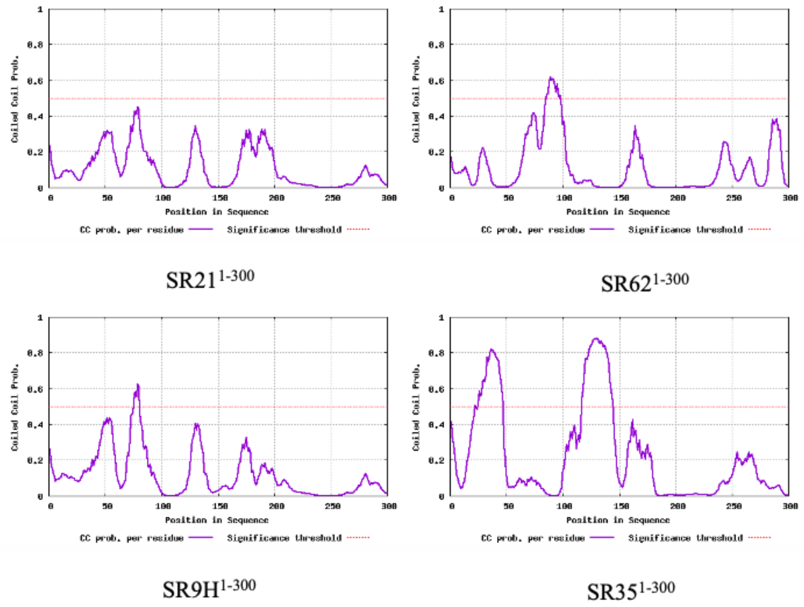

Supplementary Figure 17. Phylogenetic relationship and Coiled-coil domain prediction between the SR21-SR9 proteins and NLR type proteins confirmed from other plant species.

a. Comparison of 129 NLR proteins from plants revealed three small clades, the SR21-SR9 clade (blue), the PM3 clade (purple), and the Main SR gene clade (yellow). The tree was rooted by the TIR type NLR protein encoded by the *Linum usitatissimum L6* rust resistance gene. The kinase protein encoded by *Sr62* was used as an outgroup. b. DeepCoil prediction showed the SR21-SR9 proteins are all lacking a predictable Coiled-coil domain at their N termini (1-300 aa), kinase protein SR62$^{1\text{-}300}$ (probability at ~ 0.6) and CNL type protein SR35$^{1\text{-}300}$ (probability over 0.8) were used as the negative and positive control, respectively.

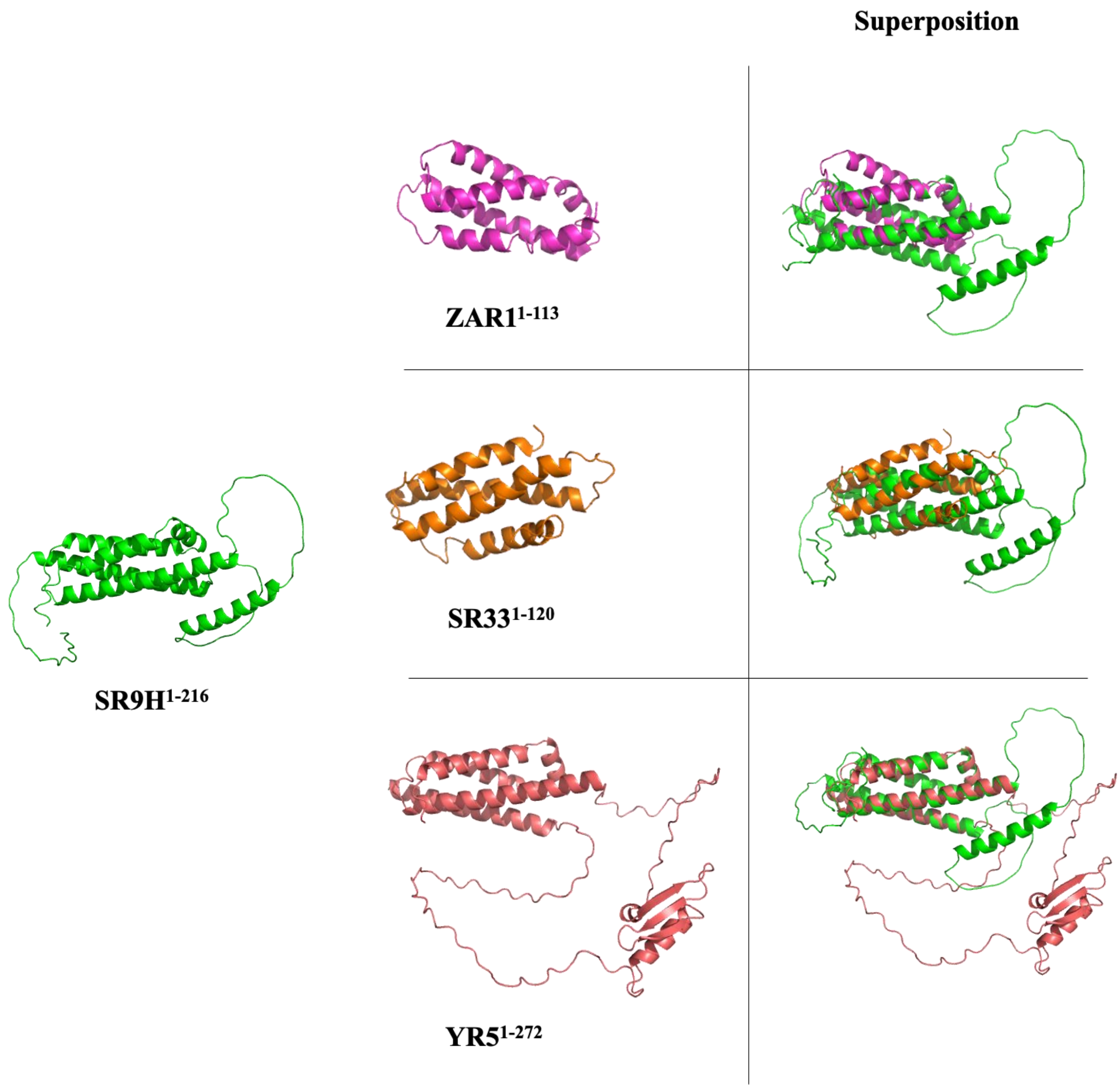

Supplementary Figure 18. Predicted SR9H N terminal region are superimposed with ZAR1, SR33, and predicted YR5 N terminal structures

Superimposed images of predicted structures of $SR9H^{1\text{-}126}$ with ZAR1 CC domain ($ZAR1^{1\text{-}113}$, 6J5W), SR33 CC domain ($SR33^{3\text{-}120}$, 2NCG), and BED domain containing N terminal fragment of YR5 ($YR5^{1\text{-}272}$, AlphaFold predicted structure), respectively.

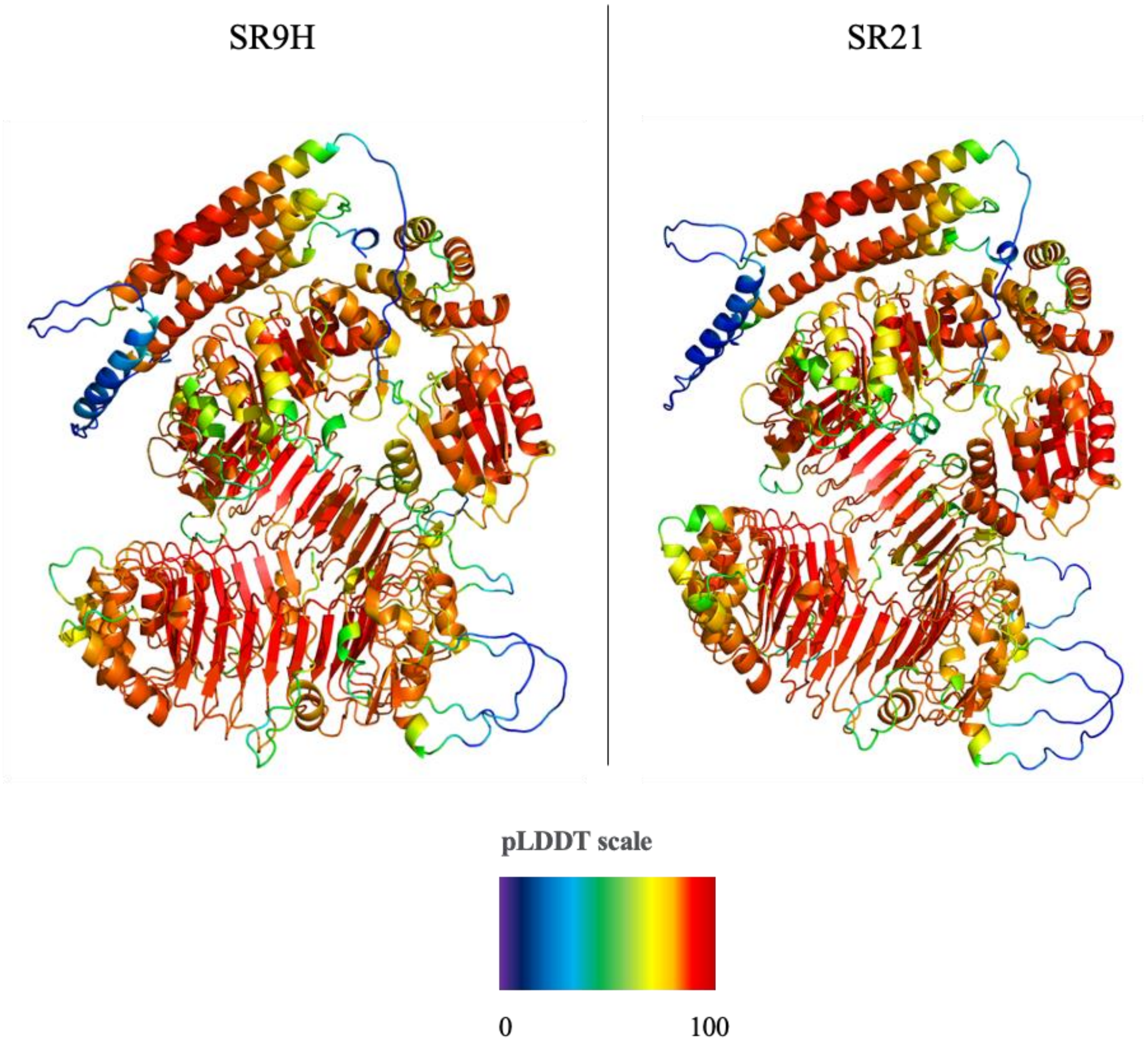

Supplementary Figure 19. SR9H and SR21 full length structures predicted by AlphaFold2 shaded with pLDDT values.

The pLDDT (predicted Local Distance Difference Test) value of the two predicted full length proteins are shaded with rainbow colour. The red colour representing high pLDDT value (high confidence) while blue colour indicating a low pLDDT value (low confidence). In a 0 to 100 scale, the pLDDT values were calculated as ranging from 23.49 to 96 for SR9H, and from 23.95 to 96.73 for SR21.
