# Supplementary material for: Single amino acid change alters specificity of the multi-allelic wheat stem rust resistance locus SR9
Source: Nat Commun. 2023 Nov 14;14:7354. doi: 10.1038/s41467-023-42747-9 (PMC10645757; doi:10.1038/s41467-023-42747-9)
Supplement: Supplementary file 3 — Description of Additional Supplementary Files [file 41467_2023_42747_MOESM3_ESM.pdf]

Description of Additional Supplementary Files

File Name: Supplementary Data 1
Description: Summary of Pgt races used in this study

File Name: Supplementary Data 2
Description: List of mutant lines used for RenSeq analysis

File Name: Supplementary Data 3
Description: SR9 alleles have unusually large number of 42 LRR units in comparison with other 11 cloned Sr proteins, and flax rust R protein L6.

File Name: Supplementary Data 4
Description: Predicted NLRs of CSv2 in the interval Chr2B:691090675-691231804

File Name: Supplementary Data 5
Description: Primers used in the present study

File Name: Supplementary Data 6
Description: Sequence polymorphism between Sr9e_h1 and Sr9e_h2

File Name: Supplementary Data 7
Description: Pgt races used to determine the resistance profiles of Sr9e_h1 and Sr9e_h2.

File Name: Supplementary Data 8
Description: Seedling infection types of 12 races of Pgt in response to wheat genetic stocks with alleles of Sr9.

File Name: Supplementary Data 9
Description: Seedling infection types of 5 races of Pgt in response to wheat lines with and without Sr9b, Sr9g, and Sr9h.

File Name: Supplementary Data 10
Description: A collection of 80 T. dicoccon accessions was used for haplotyping

File Name: Supplementary Data 11
Description: Protein polymorphisms among Sr9e haplotypes
