# Supplementary material for: Single amino acid change alters specificity of the multi-allelic wheat stem rust resistance locus SR9
Source: Nat Commun. 2023 Nov 14;14:7354. doi: 10.1038/s41467-023-42747-9 (PMC10645757; doi:10.1038/s41467-023-42747-9)
Supplement: Supplementary file 15 — Reporting Summary [file 41467_2023_42747_MOESM15_ESM.pdf]

nature portfolio

Corresponding author(s): Evans Lagudah, Matthew Rouse, Peng Zhang

Last updated by author(s): Aug 16, 2023

Reporting Summary

Nature Portfolio wishes to improve the reproducibility of the work that we publish. This form provides structure for consistency and transparency in reporting. For further information on Nature Portfolio policies, see our Editorial Policies and the Editorial Policy Checklist.

Statistics

For all statistical analyses, confirm that the following items are present in the figure legend, table legend, main text, or Methods section.

n/a	Confirmed	
☐	☒	The exact sample size (*n*) for each experimental group/condition, given as a discrete number and unit of measurement
☐	☒	A statement on whether measurements were taken from distinct samples or whether the same sample was measured repeatedly
☒	☐	The statistical test(s) used AND whether they are one- or two-sided<br>*Only common tests should be described solely by name; describe more complex techniques in the Methods section.*
☒	☐	A description of all covariates tested
☒	☐	A description of any assumptions or corrections, such as tests of normality and adjustment for multiple comparisons
☐	☒	A full description of the statistical parameters including central tendency (e.g. means) or other basic estimates (e.g. regression coefficient) AND variation (e.g. standard deviation) or associated estimates of uncertainty (e.g. confidence intervals)
☒	☐	For null hypothesis testing, the test statistic (e.g. *F*, *t*, *r*) with confidence intervals, effect sizes, degrees of freedom and *P* value noted<br>*Give P values as exact values whenever suitable.*
☒	☐	For Bayesian analysis, information on the choice of priors and Markov chain Monte Carlo settings
☒	☐	For hierarchical and complex designs, identification of the appropriate level for tests and full reporting of outcomes
☒	☐	Estimates of effect sizes (e.g. Cohen's *d*, Pearson's *r*), indicating how they were calculated

Our web collection on statistics for biologists contains articles on many of the points above.

Software and code

Policy information about availability of computer code

Data collection	Average pustule size was determined for each leaf by ASSESS 2.0
Data analysis	CLC Genomics Workbench v10.0 (Qiagen, Hilden, Germany) (https://digitalinsights.qiagen.com/products-overview/ discovery-insights-portfolio/analysis-and-visualization/qiagen-clc-genomics-workbench/)<br>CLC Sequence Viewer v8.0 (https://resources.qiagenbioinformatics.com/manuals/clcsequenceviewer/current/index.php?manual=Introduction_CLC_Sequence_Viewer.html)<br>MutantHunter pipeline (https://github.com/steuernb)<br>MuTrigo Python package (https://github.com/TC-Hewitt/MuTrigo)<br>MYbaits protocol and the Triticeae NLR bait libraries (https://github.com/steuernb/MutantHunter/blob/master/Triticea_RenSeq_Baits_ V3. fasta.gz)<br>BLASTn and SAMtools version 1.9.0<br>ITOL (https://itol.embl.de) v6<br>DeepCoil (toolkit.tubingen.mpg.de)<br>AlphaFold 2.0<br>T-Coffee program (http://tcoffee.crg.cat/apps/tcoffee/do :expresso) version :Expresso AnalySIS LS Research v2.2

For manuscripts utilizing custom algorithms or software that are central to the research but not yet described in published literature, software must be made available to editors and reviewers. We strongly encourage code deposition in a community repository (e.g. GitHub). See the Nature Portfolio guidelines for submitting code & software for further information.

Data

Policy information about availability of data

All manuscripts must include a data availability statement. This statement should provide the following information, where applicable:

- Accession codes, unique identifiers, or web links for publicly available datasets
- A description of any restrictions on data availability
- For clinical datasets or third party data, please ensure that the statement adheres to our policy

All tools and codes used in this study are listed in the the data analysis software and tools section in this form and described in the materials and methods in the main text of the manuscript. Data that support the findings of this study are openly available in NCBI, annotated genomic sequences of Sr9b, Sr9g, Sr9e_h1, Sr9e_h2, and Sr9h have been deposited at NCBI GenBank with accession numbers OP219803 (Sr9b) https://www.ncbi.nlm.nih.gov/nuccore/OP219803.1/, OP219804 (Sr9g) https://www.ncbi.nlm.nih.gov/nuccore/OP219804.1/, OP219805 (Sr9e_h1) https://www.ncbi.nlm.nih.gov/nuccore/OP219805.1/, OP219806 (Sr9e_h2) https://www.ncbi.nlm.nih.gov/nuccore/OP219806.1/, and OP219802 (Sr9h) https://www.ncbi.nlm.nih.gov/nuccore/OP219802.1/.

Human research participants

Policy information about studies involving human research participants and Sex and Gender in Research.

Reporting on sex and gender	N/A
Population characteristics	N/A
Recruitment	N/A
Ethics oversight	N/A

Note that full information on the approval of the study protocol must also be provided in the manuscript.

Field-specific reporting

Please select the one below that is the best fit for your research. If you are not sure, read the appropriate sections before making your selection.

☒ Life sciences ☐ Behavioural & social sciences ☐ Ecological, evolutionary & environmental sciences

For a reference copy of the document with all sections, see nature.com/documents/nr-reporting-summary-flat.pdf

Life sciences study design

All studies must disclose on these points even when the disclosure is negative.

Sample size	All the information regarding sample size were specifically described in the manuscript. Three biological replicates were used for qRT-PCR expression and infiltration which has been well documented as best practice for gene and protein expression studies.
Data exclusions	No data were excluded from the analyses.
Replication	Mutant plants were confirmed by at least three rounds of independent phenotyping across three generations
Randomization	When phenotyping plants, we randomized mutant and wild-type controls as is normal practice It is not relevant to other experiments as no subjects were allocated to experimental groups
Blinding	Blinding was performed when phenotyping the mutant lines for their resistance to Pgt, candidate gene confirmation by wheat transformation, rust phenotyping , qRT-PCR for determining transgene expression. It is not performed when carrying out RenSeq, Sequencing analysis, phylogenetic analysis, protein structure predictions as required by the experiments that are mainly for information exploring.

Reporting for specific materials, systems and methods

We require information from authors about some types of materials, experimental systems and methods used in many studies. Here, indicate whether each material, system or method listed is relevant to your study. If you are not sure if a list item applies to your research, read the appropriate section before selecting a response.

Materials & experimental systems

n/a	Involved in the study
☐	☒ Antibodies
☒	☐ Eukaryotic cell lines
☒	☐ Palaeontology and archaeology
☒	☐ Animals and other organisms
☒	☐ Clinical data
☒	☐ Dual use research of concern

Methods

n/a	Involved in the study
☒	☐ ChIP-seq
☒	☐ Flow cytometry
☒	☐ MRI-based neuroimaging

Antibodies

Antibodies used	Anti-GFP antibody. (mouse IgG1, clones 7.1 and 13.1) 1:2000 dilution
Validation	Anti-GFP is tested for functionality and purity relative to a reference standard to confirm the quality of each new reagent preparation. Anti-GFP mouse monoclonal antibodies are>95%pure as determined by SDS-PAGE and ion exchange HPLC analyses. Website http://www.sigmaaldrich.com/AU/en/product/roche/11814460001.
